# A Genome-Wide Association Study Reveals Dominance Effects on Number of Teats in Pigs

**DOI:** 10.1371/journal.pone.0105867

**Published:** 2014-08-26

**Authors:** Marcos S. Lopes, John W. M. Bastiaansen, Barbara Harlizius, Egbert F. Knol, Henk Bovenhuis

**Affiliations:** 1 TOPIGS Research Center IPG B.V., Beuningen, the Netherlands; 2 Wageningen University, Animal Breeding and Genomics Centre, Wageningen, the Netherlands; University of Queensland, Australia

## Abstract

Dominance has been suggested as one of the genetic mechanisms explaining heterosis. However, using traditional quantitative genetic methods it is difficult to obtain accurate estimates of dominance effects. With the availability of dense SNP (Single Nucleotide Polymorphism) panels, we now have new opportunities for the detection and use of dominance at individual loci. Thus, the aim of this study was to detect additive and dominance effects on number of teats (NT), specifically to investigate the importance of dominance in a Landrace-based population of pigs. In total, 1,550 animals, genotyped for 32,911 SNPs, were used in single SNP analysis. SNPs with a significant genetic effect were tested for their mode of gene action being additive, dominant or a combination. In total, 21 SNPs were associated with NT, located in three regions with additive (SSC6, 7 and 12) and one region with dominant effects (SSC4). Estimates of additive effects ranged from 0.24 to 0.29 teats. The dominance effect of the QTL located on SSC4 was negative (−0.26 teats). The additive variance of the four QTLs together explained 7.37% of the total phenotypic variance. The dominance variance of the four QTLs together explained 1.82% of the total phenotypic variance, which corresponds to one-fourth of the variance explained by additive effects. The results suggest that dominance effects play a relevant role in the genetic architecture of NT. The QTL region on SSC7 contains the most promising candidate gene: *VRTN*. This gene has been suggested to be related to the number of vertebrae, a trait correlated with NT.

## Background

Dominance effects are non-additive effects due to the interaction between alleles at the same locus. In livestock and plant breeding, the main benefits of dominance effects are expected in crossbreeding, since dominance has been suggested as one of the genetic mechanisms explaining heterosis [1]–[5]. However, estimates of dominance effects have not been widely used in livestock breeding because it is difficult to estimate these effects accurately based on pedigree [6].

The development of dense SNP (Single Nucleotide Polymorphism) panels offered new opportunities for detection and use of dominance at individual loci. However, genomic selection or genome-wide association studies (GWAS) mainly focused on additive genetic effects and ignored dominance. Recently, a number of studies investigated the importance of non-additive effects in genomic prediction [6]–[9] and GWAS [10], [11], showing that accounting for these effects increased the accuracy and reduced the bias of genomically-predicted breeding values in comparison to an additive model [6]–[9]. Su *et al*. [9] showed that in a purebred Duroc population the dominance variance accounted for 6% of the total phenotypic variance in daily gain, emphasizing the relevance of dominance.

Significant dominance effects on number of piglets born alive and litter size were identified in a GWAS [10]. In cattle, significant dominance effects were reported for milk production traits [11]. In both studies, additive and dominance effects were tested for each SNP using multiple regression, i.e. this approach simultaneously tested for the significance of the SNP and investigated its mode of gene action. An alternative way of testing for additive and dominance effects of a SNP consists of two steps: 1) SNP genotypes are fitted in the model as a class variable and the significance of a genetic association is tested, irrespective of the mode of gene action and subsequently, 2) only the SNPs with a significant genetic effect are tested for their mode of gene action. This two-step approach is favored over the multiple regression model because a single class variable is used to capture the total genetic variation that is explained by the SNP, while the multiple regression method applied by Coster *et al.* [10] and Boyesen *et al.* [11] will divide the variation over two parameters which are then separately tested for significance. In addition, the multiple regression model requires approximately twice the number of tests that are performed by the two-step approach. Therefore, for certain modes of gene action, this multiple regression model leads to a reduction of power. Fitting a SNP as a class variable has been successfully applied in previous GWAS [12]–[14]. However, in these studies the mode of gene action of the significant SNPs was not evaluated.

In QTL mapping studies in pigs, number of teats (NT) has been one of the most extensively studied traits. NT is an important trait for breeding programs because the number of piglets in a litter is often larger than the number of functional teats of the sow due to the remarkable improvement in sow prolificacy over the last decades [15]. A lower NT than the number of piglets induces suckling competition, which can lower pre-weaning growth and survival. Previous linkage studies on NT [16]–[26] have shown evidence of both additive and dominance effects on this trait. These studies applied low density microsatellite panels to relatively small experimental crosses, resulting in the identification of QTL with wide confidence intervals. The use of dense SNP panels using a GWAS gives the opportunities to narrow down the QTL regions in purebred populations.

The aim of this study was to detect additive and dominance effects on number of teats, specifically to investigate the importance of dominance using a high-density SNP panel in a Landrace-based population of pigs.

## Methods

### Ethics Statement

The data used for this study were obtained as part of routine data recording in a commercial breeding program. Samples collected for DNA extraction were only used for routine diagnostic purpose of the breeding program. Data recording and sample collection were conducted strictly in line with the Dutch law on the protection of animals (Gezondheids- en welzijnswet voor dieren).

### Genotypes

DNA from 1,795 animals was extracted from blood, hair follicles or ear tissue. Genotyping was performed using the Illumina 60K+SNP Porcine Beadchip [27]. Positions of the SNPs were based on the Pig genome build10.2 [28]. The first step of the quality check consisted of excluding SNPs with GenCall score <0.15, with unknown position on the build10.2 [30] and SNPs located on both sex chromosomes. Based on these criteria 8,990 SNPs were excluded from the data. Further, 13,315 SNPs were excluded because they failed at least one of the following criteria: call rate <0.95, minor allele frequency <0.01 and/or strong deviation from Hardy Weinberg Equilibrium (χ^2^ values>600). Finally, 9,016 SNPs were excluded because a genotype class had a frequency <0.02. This last step was necessary because this study focused on both additive and dominance effects and therefore observations were necessary in all three genotype classes. After these quality checks, 32,911 out of 64,232 SNPs were used for the GWAS.

In total, 71 individuals with missing genotype frequency >0.05 (based on 32,911 SNPs that passed the quality check) were excluded. In addition, animals that had at least one of their parents genotyped were checked for pedigree inconsistencies. The parental check consisted of comparing the genotypes of the offspring and their parents (one or both parents) at all loci. If a Mendelian inconsistency was detected (e.g. offspring genotype = *BB* and parent genotype = *AA*), the genotype of the offspring at that specific locus was set to missing. Further, if the proportion of Mendelian inconsistencies was >0.01, either a pedigree mistake or a mistake during the genotyping process was assumed and the offspring was excluded from the data set. If the proportion of Mendelian inconsistencies was >0.01 for all offspring of a given parent, the parent was excluded as well, however, this was not observed in the current data set. A total of 17 animals (offspring) were excluded based on the described procedure. A further 68 animals were excluded because their NT was not recorded. Finally, 89 animals were excluded because they were the unique observation from their herd-year-season class, leaving 1,550 genotyped and phenotyped animals for this study.

### Animals and Phenotypes

The evaluated population consisted of 630 males and 920 females from a Landrace-based line. These animals were born between 2005 and 2012 on 30 different farms. A total of 952 genotyped animals had at least one of their parents genotyped as well. The group of genotyped parents consisted of 138 sires and 145 dams. The NT of each individual was counted at birth as part of standard data recording in a commercial breeding program. Only the total NT was counted. The number of left and right teats, and teat malformations was not recorded. The average NT in the dataset was 15.61±1.05, ranging from 12 to 20 teats. The dataset used in this study is available upon request. Contact Egbert Knol by e-mail: Egbert.Knol@TOPIGS.com.

### Association Analyses

A single-SNP GWAS for additive and dominance effects on NT was performed using an animal model. To capture both additive and dominance contributions to the variance explained by a SNP in a single model parameter, the genotypes were fitted as a class variable with three levels. The following model was used:
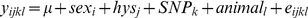
(1)where *y_ijkl_* was the phenotype of animal *l*; *µ* is the overall mean; *sex_i_* was the fixed effect of sex *i*; *hys_j_* was the fixed effect of the herd (h) year (y) season (s) *j* of birth (*j* = 1 to 291); *SNP_k_* was the SNP genotype *k* (AA, AB or BB) fitted as a fixed effect; *animal_l_* was the random additive genetic effect which was assumed to be distributed as ∼*N*(0, ***G
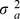
***), which accounted for the (co) variances between animals due to genomic relationships by formation of a *G* matrix (genomic relationship matrix); and ***e_ijkl_*** was the random residual effect which was assumed to be distributed as ∼*N*(0, **I
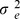
**). Variance components were re-estimated in each SNP association analysis. The analyses were performed using ASReml v3.0 [29].

The *G* matrix was used to account for genomic relationships and to reduce the risk of false-positive associations due to population stratification and was computed as described by VanRaden [30];
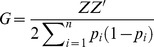
where *Z* is a matrix that contains all SNP genotypes of all animals corrected for the allele frequency per SNP; *n* is the total number of SNPs present in *Z* and *p_i_* is the frequency of the allele B of SNP *i*. The SNP genotypes were coded as 0, 1 and 2, being 0 = *AA*, 1 = *AB* and 2 = *BB*. Allele frequencies of the current sample were used in the calculations to obtain *Z* and *p_i_*.

Residuals were visually inspected for normality based on a QQ-plot of the residuals from model (1) without a SNP effect, using the qqnorm() function in R [31]. The inflation factor (lambda) for the distribution of *P*-values from the GWAS was estimated using the estlambda() function of the R package GenABEL [32]. A genome-wide False Discovery Rate (FDR) was applied using the R package qvalue [33] to avoid false positives due to multiple testing. An FDR ≤0.10 was used to indicate significant association.

All significant SNPs located within 5 Mb from another significant SNP were considered to belong to the same QTL region. When more than one QTL region was detected on the same chromosome, linkage disequilibrium (LD) was used to assess the dependence of these effects. If the LD (r^2^) of all SNP-pairs between the two different regions was <0.70, these regions were considered independent. LD estimates were obtained using Haploview v4.2 [34].

The total variance explained by each QTL (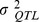) was estimated as the sum of its additive (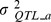) and dominance (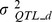) variances, which were estimated as follows:
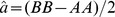


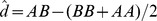


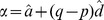


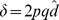


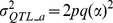


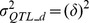


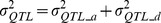
where *p* and *q* are the allele frequencies, 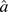 the additive and 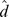 the dominance effects estimated from the genotype effects (*AA*, *BB* and *AB*) of the most significant SNP in a QTL region, 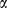 is the allele substitution effect and 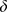 is the dominance deviation. The QTL variance was expressed as a fraction of the total phenotypic variance (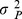, being the summation of the additive and environmental variances) which was estimated based on model (1) without a SNP effect.

### Testing for additive and dominance effects

To determine if the SNP had a significant additive effect, dominance effect or both, contrasts for additive and dominance effects were tested for the most significant SNP in each QTL region. Testing was performed using the option !CONTRAST in ASReml v3.0 29] in model (1). Additive effects were declared when the contrast between the effects of the two homozygous genotypes was significantly different from zero (*P*<0.01). Dominance effects were declared when the contrast between the average effect of the two homozygous genotypes (*AA* and *BB*) and the effect of the heterozygous genotype was significantly different from zero (*P*<0.01).

Results from the current study were compared with previously identified QTL using the alignment of genetic and physical maps in PigQTLdb [35]. Genes located in QTL regions, including flanking regions of 0.2 Mb upstream or downstream of QTL regions, were considered as candidates. Gene searches were carried out with NCBI map viewer (http://www.ncbi.nlm.nih.gov/projects/mapview/map_search.cgi?).

## Results

The additive genetic variance for NT estimated using model (1) without a SNP effect was 0.43 and the corresponding heritability was 0.37±0.05. The estimated effects for sex showed that males presented 0.35±0.09 more teats than females. Although NT is a count variable, the residuals follow a normal distribution (Figure S1).

**Figure 1:**
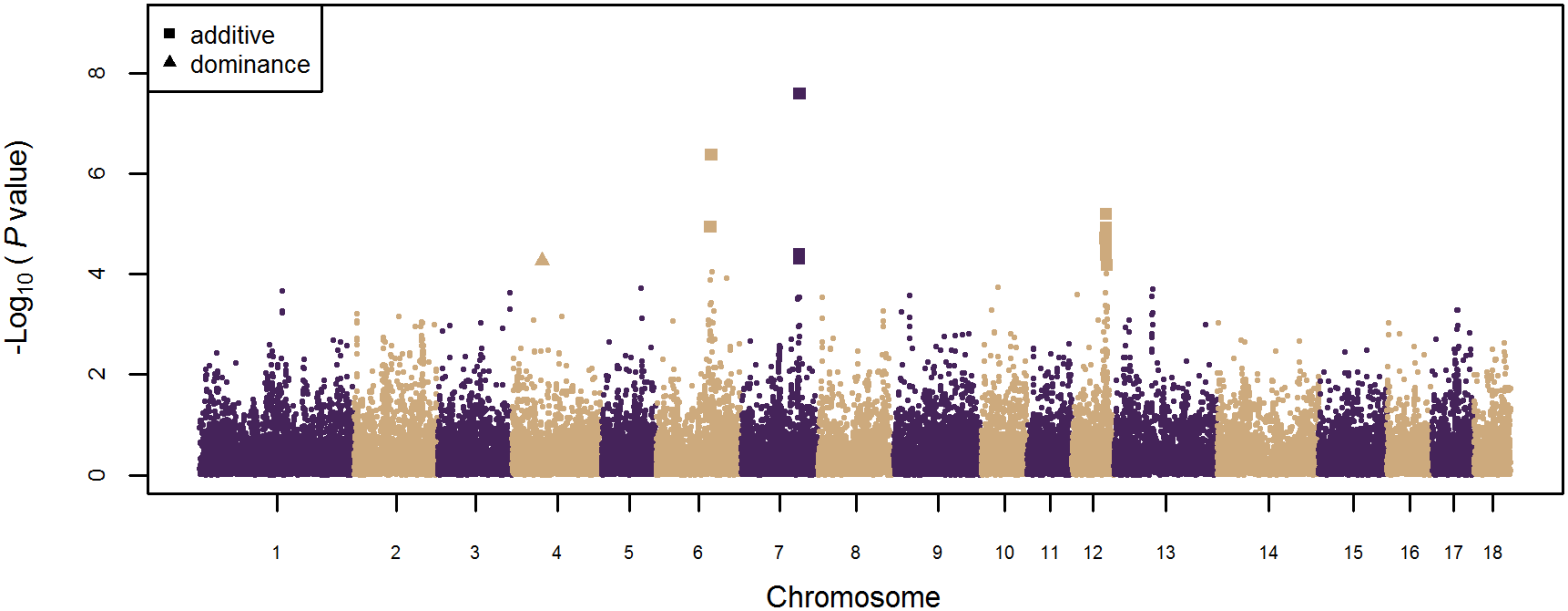
Genome-wide association study for additive and dominance effects on number of teats in pigs. On the y-axis is the −log10 (*P*-values) of single-SNP association with number of teats in pigs. On the x-axis is the physical position of the SNPs across the 18 autosomes. SNPs associated (false discovery rate ≤0.10) with number of teats having additive and dominance effects are represented by squares and triangles, respectively.

An inflation factor of 1.13 was estimated, indicating that any major effects of population stratification were accounted for in the analyses. In total, 21 SNPs were associated with NT (Figure 1). These SNPs were located in four different QTL regions on SSC4, 6, 7 and 12 (Table 1).

**Table 1:** Characterization of the QTL regions.

SSC^1^	Position (Mb)			Most sign. SNP	MAF	MGF	−log_10_ (*P*value)	SNP effects^2^		SNP variance (% of 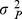 )	
	Start		End					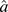	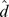	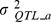	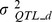
4	44.53	-	44.53	ASGA0019540	0.31	0.09	4.27	0.04	−0.26*	0.13	1.14
6	101.77	-	104.41	ALGA0036369	0.36	0.14	6.37	0.27*	−0.18	1.97	0.60
7	103.03	-	103.59	ASGA0035500	0.32	0.12	7.59	0.29*	0.04	3.02	0.02
12	52.71	-	54.68	ALGA0120076	0.43	0.20	5.19	0.24*	0.05	2.25	0.06
**Total**										**7.37**	**1.82**

Minor allele frequency (MAF), minor genotypic frequency (MGF), −log10 (P-values) of the association analysis, additive (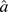) and dominance (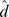) effect estimates, proportion of the total phenotypic variance (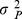) explained by the additive and dominance variances (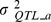 and 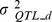) based on the most significant SNP of each QTL region.

1 SSC: *Sus scrofa* chromosome;

*Contrast to evaluate the mode of gene action was significant (P<0.01);

2 Additive effects expressed in absolute values.

One QTL region was characterized as dominant and three as additive. Estimated effects for QTLs that were characterised as showing additive gene action ranged from 0.24 to 0.29 teats (in absolute values). The QTL that was characterised as showing dominant gene action showed a negative dominance effect (−0.26 teats). The summation of 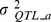 of all four QTLs corresponds to 7.37% of 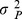 and 23.25% of the additive genetic variance. The summation of 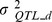 of all four QTLs corresponded to 1.82% of 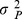, which is one-fourth of the variance explained by additive effects.

### Additive QTL

The QTL region on SSC6 contained two SNPs with significant associations. This QTL region was located between 101.77 and 104.42 Mb and ALGA0036369 was the most significant SNP with −log_10_ (*P*-value) of 6.37. This SNP showed an additive effect of 0.27 teats and a dominance effect of −0.18 teats. However, only the contrast for additive effects was significant for this SNP.

On SSC7, between 103.03 and 103.59 Mb, the highest GWAS peak was found for SNP ASGA0035500 with a −log_10_ (*P*-value) of 7.59. This SNP showed an additive effect of 0.29 teats, a dominance effect of 0.04 teats and explained 3% of the phenotypic variance.

On SSC12, between 52.71 and 54.68 Mb, was located the third most significant QTL region which was also the region characterized by the largest number of significant SNPs in this study (15 SNPs). The most significant SNP in this region (ALGA0120076) showed an additive effect of 0.24 teats and a dominance effect of 0.05 teats.

### Dominant QTL

The SNP ASGA0019540 located at 44.53 Mb on SSC4 was the only significant marker in this QTL region. This SNP showed a dominance effect of −0.26 teats and its 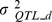 corresponded to 1% of the total 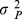 (Table 1). The additive effect for this QTL was not significant (*P*>0.01) and thus, the mode of gene action of this QTL seems purely dominant. This SNP presented a minor allele frequency of 0.31 and a minor genotypic frequency of 0.09 (Table 1), indicating that each genotype class consisted of a considerable number of observations.

## Discussion

### QTL and candidate genes

The majority of studies that use genomic information in livestock species have been directed towards discovery or use of additive genetic effects. Such studies are generally performed by applying a linear regression to obtain SNP allele substitution effects. In the current study, SNP genotypes were fitted in the model as a class variable. Using this approach, and basically the same data structure typically used in association studies, it was possible to distinguish additive and dominance genetic effects.

In the present study, four QTL regions related to NT were identified. Among these QTLs, three presented significant additive effects, while one only showed significant dominance effect. The proportion of the total phenotypic variance explained by the additive effects were also higher compared to the proportion explained by dominance effects, being respectively, 7.37 and 1.82% of 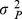 (Table 1). Although these percentages were likely overestimated due to the Beavis effect [36], which especially has an impact when the effects of a SNP are small, these results present convincing evidence that dominance plays a role in the genetic architecture of NT. These results also suggest that additive effects contribute more to the genetic variance of NT than dominance effects. In pigs, other authors have also demonstrated that additive effects contribute more to the genetic variance of traits than dominance effects. Su *et al*. [9] showed that additive genetic variance of daily gain was 3.73 fold higher than the dominance genetic variance. Recently, Nishio *et al*. [37] demonstrated for a number of traits in pigs that the contribution of additive effects to the genetic variance was 18–31% higher than the contribution of dominance effects.

All QTL regions identified in this study overlap with QTL regions that have been detected previously in one or more studies [17], [20], [21], [24]–[26]. However, this study is the first to describe a dominant QTL effect on SSC4. On this chromosome, previous studies [24], [25] have shown QTLs with additive effects. In addition, the length of the QTL regions in this study has been considerably reduced. For example, the most significant QTL in this study (SSC7) showed significant associations in the region between 103.03 and 104.35 Mb (length of the region is 1.32 Mb). Guo *et al*. [24] reported a QTL related to NT on SSC7 with a confidence interval of 112 cM (∼112 Mb).

**Figure 2:**
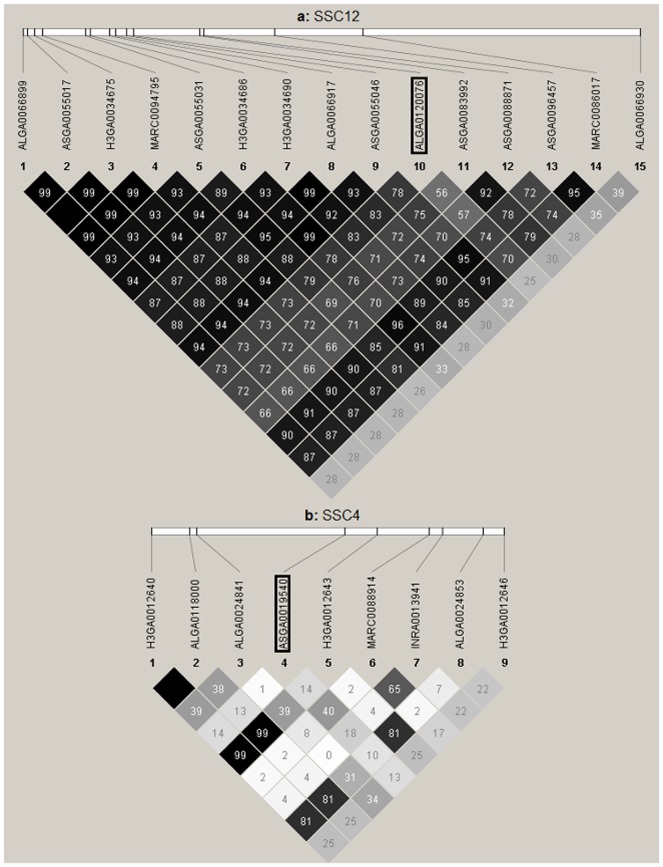
Difference in linkage disequilibrium (LD) between two distinct QTL regions. (a) LD (*r2*) between the significant SNPs of the QTL region on *Sus Scrofa* chromosome (SSC) 12; the most significant SNP in this region is surrounded by a square. (b) LD between the SNPs located 0.2 Mb downstream and upstream of the only significant SNP (surrounded by a square) of the QTL region on SSC4. The numbers inside the diamonds are the LD measurements (*r^2^*) on a scale of 0 to 100%.

The QTL region on SSC4 contained only a single significant SNP while the region on SSC12 contained 15 significant SNPs. The QTL region on SSC12 covered 1.97 Mb and the average LD (*r^2^*) between the 15 SNPs was 0.78 (Figure 2) and the smallest pairwise *r^2^* between SNPs in this region was 0.56, except for the most distal SNP. The average LD between the significant SNP and the neighbouring SNPs (within 0.2 Mb) in the region on SSC4 (9 SNPs) was very low (0.15). The low LD between SNPs in this region, and with the single significant SNP in particular, explains why the significant associations could not be confirmed by significant associations of neighbouring SNPs with NT. An alternative explanation for observing only one single significant SNP on SSC4 could be that this SNP was misplaced in the Pig genome build10.2 [28]. However, Pearson correlations (r) between this SNP (genotypes coded as 0, 1 and 2) and all other SNPs used in the GWAS (across the whole-genome) showed that the highest correlations were found with SNPs, who according to the Pig genome build10.2, should be considered its neighbouring SNPs (data not shown). Therefore, there is no evidence suggesting that the location of this SNP is wrong. Thus, it was concluded that although the QTL on SSC4 is only picked up by a single SNP, this QTL is probably not an artefact. However, the effect of this QTL region needs to be confirmed based on independent studies.

To distinguish between additive and dominance effects, observations are necessary for all three genotype classes. Therefore, a total of 9,016 SNPs with minor genotypic frequency <0.02 were excluded. The lowest minor genotypic frequency of a significant SNP observed in the current study was 0.09 (143 out of 1,550 individuals) for the dominant QTL in the region SSC4. As the proportion of SNPs excluded based on their minor genotypic frequency was relatively high, an additional analysis was performed to investigate whether any of these 9,016 excluded SNPs were associated with NT, even though these SNPs do not allow the investigation of the mode of gene action. For this analysis, the least frequent genotype class of these SNPs was set to missing and SNPs with two genotype classes <0.02 were not evaluated (n = 115). None of these SNPs showed a significant association (FDR<0.10).

The region detected on SSC7 has been identified as a QTL for NT in other populations [16], [21], [22], [24], [25], as well as a QTL for carcass length [38]–[40] and number of vertebrae and ribs [41]–[45]. A phenotypic correlation of 0.24 between NT and number of thoracic vertebrae has been estimated [42], and a larger number of vertebrae is associated with an increase in carcass length and number of ribs [46], [47]. Thus, the region is of great interest for pig breeders with favourable pleiotropic effects on economically important traits, including mothering ability of the sows due to the increase in NT, and increased pork production per animal due to longer carcasses.

The Vertnin *(VRTN)* gene appeared as the most promising candidate in this region. *VRTN* encodes a potential DNA binding factor and has been described as an essential factor for development of the embryo in different species [48]. Due to its biological function, this gene has been indicated as a candidate gene for number of vertebrae [42], [48], [49]. Recently, Fan *et al*. [49] performed a fine mapping study aiming to identify the causal mutation of a QTL for number of vertebrae in the same region. By applying an identity-by-descendent sharing method, the QTL region was narrowed down to a 128 Kb region that harboured the *VRTN* gene. The region was defined by two SNPs: ASGA0035500 and INRA0027623, which were, respectively, the first and the third most significant SNPs for NT in the current study. Later, Fan *et al*. [49] identified a possible causal mutation in the *VRTN* gene. Due to the positive relation between NT and number of vertebrae and the similarities between the results on SSC7 of the present study and the results of Fan *et al.* [49], it can be assumed that the *VRTN* gene may also have an effect on NT.

In the other QTL regions, no obvious genes that could effectively affect NT were identified. The relationship between *VRTN* and NT needs to be further investigated in order to validate the effect of this gene on the genetic architecture of NT.

### Implications

The term heterosis was coined by Shull [50] to describe an improved performance of crossed individuals compared to the average performance of their parental inbred lines. However, the performance of crossbreds depends partly on the degree and sign of the dominance effects of the loci affecting the trait [51], [52], which can also lead to negative heterosis (crossbreds performing worse than the average of their parents). Thus, the definition of heterosis being the deviation of crossbred performance compared to the average performance of the two parental breeds [53] is more appropriate.

In pigs, negative heterosis (also called outbreeding depression or hybrid inferiority) has not often been reported in the literature. Bereskin *et al*. [53] showed that crossbred pigs on average had higher levels of backfat and lower levels of ham and loin percentage than their purebred parents. However, more examples of negative heterosis have been published in other species. In Drosophila, negative heterosis has been identified for the degree of deficient venation [54] and in an F1 chicken population, negative heterosis was reported for leukocyte ratio at 8 weeks of age [55]. Minozzi *et al.* [56] reported negative direct heterosis for general immune response traits in White Leghorn chickens. Barbato [57] observed negative heterosis for abdominal fat in chickens. Denic *et al.* [58] described that negative heterosis in humans is related to higher rates of breast and ovarian cancer.

In the current study, the dominant QTL identified on SSC4 showed negative estimate for dominance effect (−0.26 teats). Based on this locus, negative heterosis would be expected for NT, assuming that dominance effects are the main cause of heterosis. However, it is important to keep in mind that the main cause of heterosis is still under debate. While it has been shown in few studies that dominance is an important factor contributing to heterosis [1]–[5], in other studies, the main cause of heterosis has been attributed to epistasis [59]–[61]. Recently, Amuzu-Aweh *et al.* [62] evaluating egg production traits in chickens, showed that although dominance cannot fully explain heterosis, a dominance model can achieve considerable accuracy of prediction of heterosis. In pigs, the genetic background of heterosis has not been elucidated. Therefore, epistatic interactions also might play a role; however, in segregating populations, the power to identify epistatic interactions between QTLs is low [60].

As a further step, the role of dominance effects on the genetic architecture of NT effects should be evaluated in a crossbred population, since non-additive effects are expected to be of importance in crossbreeding [63], [64]. Nonetheless, the results of this study showed that dominance effects explain an important fraction of the phenotypic variance even in a purebred population.

**Figure 3:**
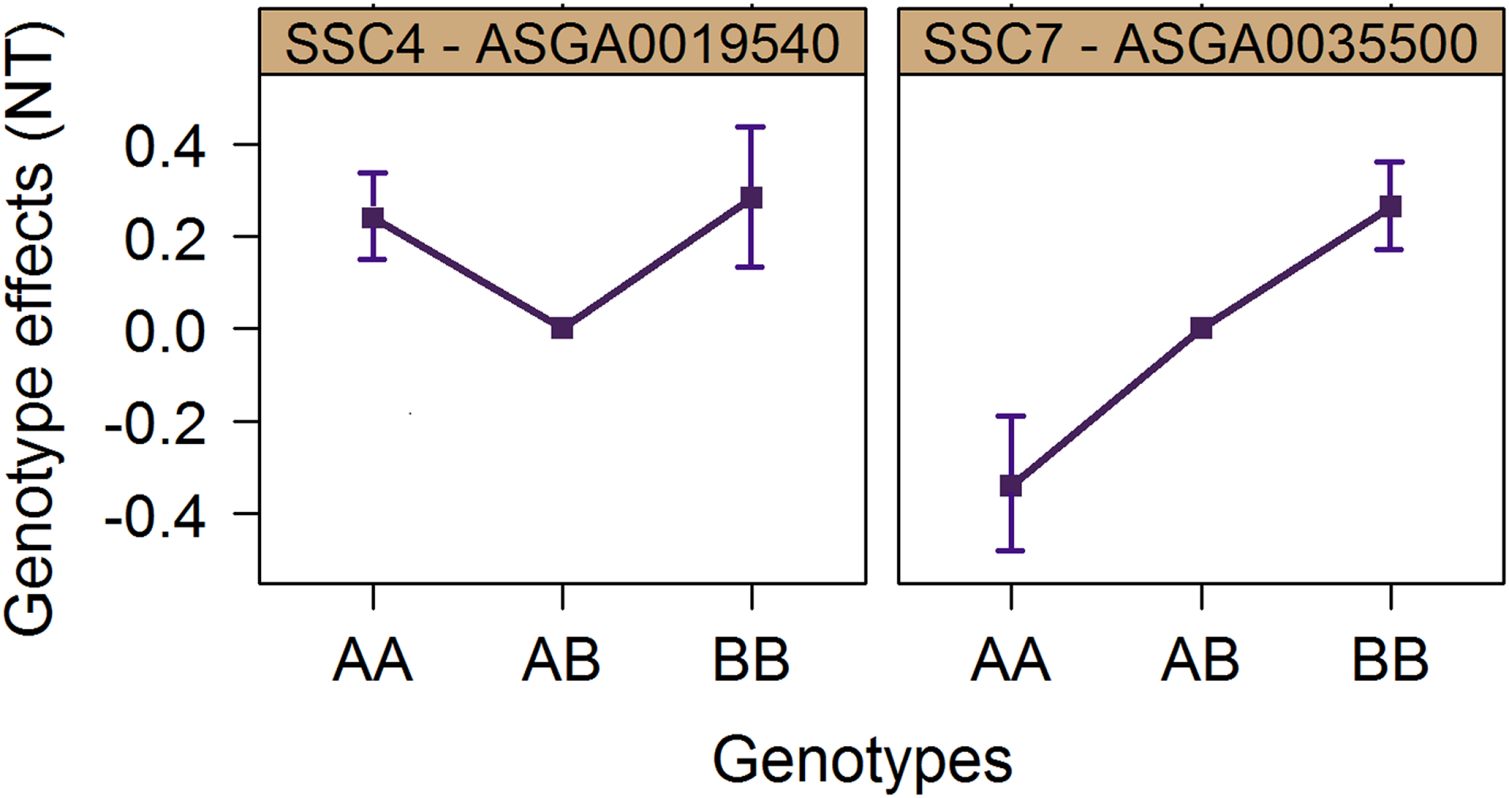
Genotype effects. Genotype effects and their standard errors of the most significant SNPs on *Sus scrofa* chromosomes (SSC) 4 and 7 on number of teats (NT). The genotypic effects are relative to the effect of the heterozygous genotype, which was set to zero.

The genotype effects of the QTL region on SSC7 (Figure 3) showed that this QTL has a clear additive effect. For such QTL, the traditional selection that is based on allele substitution effects would be sufficient, as selection for higher NT would lead to the fixation of the favourable allele B (ignoring the potential impact of drift).

A more challenging situation is encountered with the QTL region on SSC4 which may require the adoption of different strategies, such as mate allocation. Applying an additive model for estimating breeding values would not be efficient, since the additive effect of this QTL is close to zero (Figure 3). In cases of overdominance, selection tends to keep heterozygotes in the population instead of fixing one of the alleles [65]. However, in order to improve the population mean, the goal for these two dominant QTL must be the fixation of one of the alleles in order to avoid heterozygous animals with their negative dominance effects. More specifically, for the QTL on SSC4, the selection should be towards the fixation of the A allele, since this is the most frequent allele (f(A) = 0.69). If selection is aimed at fixation of the B allele, it would take longer before this allele becomes fixed, and in the meantime an increase in the frequency of AB animals would be observed, negatively affecting the mean NT of the population. Finally, when this QTL presents the same effect on different lines, all lines within a breeding program should be fixed for the same allele in order to maximize the performance of crossbred animals.

According to Toro and Varona [7], it is easier to include dominance effects in genomic evaluations compared to including them in the traditional selection using pedigree information. These authors concluded that the use of dominance effects in a scenario of genomic selection increases the accuracy of estimated breeding values and still offers the opportunity of applying mate-allocation. Wang *et al*. [65] described that the genetic progress of traits controlled only by additive genetic effects will generally achieve the target genotype faster than traits with considerable overdominance. Although the genetic progress is slower in the presence of dominance compared to the situation when only additive effects play a role, if dominance effects exist and are not properly taken into account, the genetic progress may be even slower.

## Conclusions

In this study, four QTLs, three additive and one dominant, were identified by applying a two-step approach; first testing for significant genetic effect and then testing for additive and/or dominant gene action only of the SNPs with significant genetic effects. In total, the 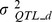 corresponded to approximately one-fourth of the variance that was explained by 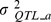, demonstrating that dominance effects play a role in the genetic architecture of NT. The QTLs with significant additive effects overlap with earlier identified QTLs, however, the QTL regions were considerably reduced in size. Selection based on these QTLs would benefit mothering ability of the sows due to the increase in NT, as well as increasing pork production of finishing pigs due to pleiotropic effects on number of vertebrae and carcass length.

## Supporting Information

Figure S1 **QQ-plot of the residuals from the linear model without a SNP effect.**
(TIFF)
