# Supplementary figures and images for: A Genome-Wide Association Study Reveals Dominance Effects on Number of Teats in Pigs
Source: PLoS One. 2014 Aug 26;9(8):e105867. doi: 10.1371/journal.pone.0105867 (PMC4144910; doi:10.1371/journal.pone.0105867)

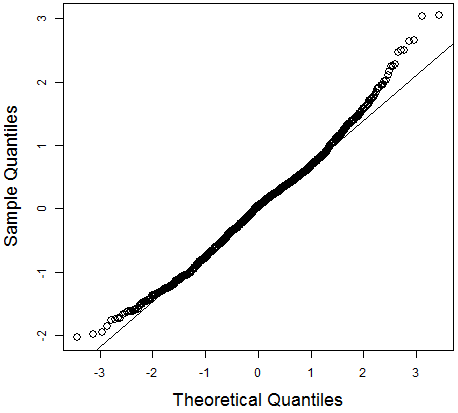

Supplement: Figure S1 — QQ-plot of the residuals from the linear model without a SNP effect. (TIFF) [file pone.0105867.s001.tiff]
